# Endocardite da Válvula Mitral – Uma Causa Rara num Doente com Neoplasia

**DOI:** 10.36660/abc.20230268

**Published:** 2023-10-20

**Authors:** Fabiana Duarte, Carina Machado, Luis Oliveira, Duarte Machado, Raquel Dourado

**Affiliations:** 1 Hospital do Divino Espirito Santo de Ponta Delgada EPE Açores Portugal Hospital do Divino Espirito Santo de Ponta Delgada EPE – Cardiologia, Açores – Portugal

**Keywords:** Endocardite, Tratamento Farmacológico, Ecocardiografia

## Introdução

Pacientes com câncer são propensos a endocardite infecciosa (EI), associada a agentes causais específicos, levando a resultados piores. A prevalência de EI em pacientes com câncer varia de 5,6% a 18%, com uma taxa de mortalidade relatada em 1 ano de quase 30%. ^1 - 3^ Fatores de risco, como sistema imunológico suprimido, uso de cateter intravascular e hipercoagulabilidade, predispõem os pacientes com malignidade à EI. ^2 , 3^

A evolução clínica é desfavorável devido às altas taxas de mortalidade relacionadas à resistência aos antibióticos, às condições médicas subjacentes, à falta de intervenções cirúrgicas realizadas e à descontinuação do regime terapêutico antitumoral. ^2 - 5^

De acordo com a literatura, espécies de estafilococos, principalmente *Staphylococcus aureus* e *Enterococos* , são microrganismos comuns causadores de EI em pacientes com câncer. ^2 , 3^

Descrevemos um caso incomum de EI da valva mitral causada por *Serratia marcescens* em paciente com malignidade hematológica. Embora a bacteremia causada por *S. marcescens* , um bacilo gram-negativo não-HACEK, não seja incomum, sua associação com EI é bastante rara. ^6 , 7^

### Relato de caso

Paciente do sexo masculino, 51 anos, com histórico de hipertensão, tabagismo e obesidade, foi submetido à penectomia parcial por carcinoma espinocelular ulcerado do pênis em janeiro de 2020. Em março de 2021, uma tomografia computadorizada (TC) torácico-abdominopélvica revelou uma massa na esquerda da região inguinal com linfonodos aumentados ilioinguinais e para-aórticos. Uma biópsia confirmou metástases linfonodais regionais (LN) de carcinoma peniano e, após discussão multidisciplinar, o paciente foi proposto para quimioterapia neoadjuvante antes da ressecção do LN. Três ciclos de quimioterapia foram concluídos através de acesso venoso central implantável de longo prazo na veia subclávia direita. Nenhuma complicação imediata foi relatada.

Aproximadamente 24 horas após o último tratamento, o paciente compareceu ao pronto-socorro com queixa de cansaço, perda de apetite e febre. O paciente negou dor pleurítica ou sintomas abdominais. Ao exame físico apresentava-se hipotenso e febril (temperatura timpânica 38,4°C), com polipneia, hipoxemia e desorientação temporal e espacial. Nenhum achado adicional foi relatado, incluindo exame cardíaco normal. A análise da amostra de sangue revelou anemia (hemoglobina 9,8 g/dL) e trombocitopenia (contagem de plaquetas 100.000/µL), lesão renal aguda (creatinina 3,68 mg/dL, ureia 130 mg/dL), rabdomiólise (creatina quinase 18.567 U/L), alta níveis de proteína C reativa (PCR 19 mg/dL) e procalcitonina (PCT 51,6 ng/mL).

**Figura 1:**
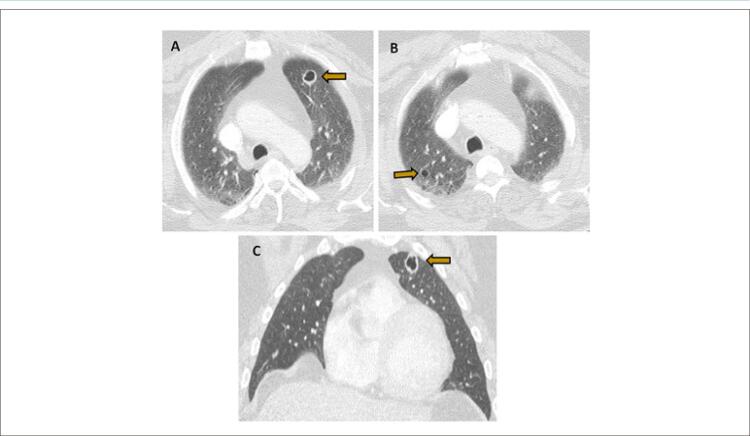
– A, B, C) Tomografia computadorizada de tórax (janela pulmonar) demonstrando lesões nodulares cuneiformes bilaterais, algumas com cavitação (seta amarela), a maioria localizada na periferia. Os nódulos têm diâmetros que variam de alguns milímetros a 18 mm, sendo o maior deles localizado no lobo superior esquerdo.

Durante a investigação diagnóstica, a tomografia computadorizada de tórax evidenciou múltiplos nódulos pulmonares sólidos e cavitários sugestivos de êmbolos pulmonares sépticos ( Figura 1 , AC). Nesta fase foi proposto o diagnóstico de choque séptico com disfunção multiorgânica. Foram coletados dois conjuntos de hemoculturas periféricas e o paciente iniciou terapia antimicrobiana empírica com ceftriaxona e metronidazol e foi internado em Unidade de Terapia Intensiva.

Sua condição piorou, necessitando de terapia vasopressora de norepinefrina em altas doses e oxigênio suplementar. Uma tomografia computadorizada abdominal/pélvica revelou progressão da doença neoplásica e uma tomografia computadorizada de crânio não mostrou sinais de embolização cerebral.

**Figura 2:**
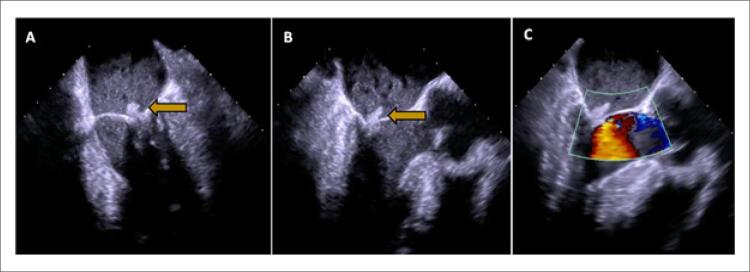
– Ecocardiografia transesofágica inicial realizada durante a internação hospitalar. Revelou vegetação mitral grande, filiforme e móvel (diâmetro máximo de 23 mm), aderida à superfície atrial do folheto mitral posterior, envolvendo regiões de vieira P2 e P3 (seta amarela); Visão longitudinal do ETE em 60º (A) e 120º (B). Não foram evidenciados prolapso de vieira ou regurgitação mitral (C).

De acordo com testes de sensibilidade a antibióticos, *Serratia marcescens* foi isolada de hemoculturas e o esquema antimicrobiano inicial foi ajustado para piperacilina-tazobactam. O cateter venoso central de longa permanência foi retirado e enviado para cultura, que isolou o mesmo microrganismo. Um ecocardiograma transtorácico foi realizado inicialmente, mas foi inconclusivo devido a uma janela acústica muito ruim. O ecocardiograma transesofágico (ETE) realizado durante a internação revelou uma única vegetação valvar mitral volumosa (diâmetro máximo de 23 mm) aderida ao folheto posterior da valva mitral nos segmentos de engrossamento P2 e P3, sem insuficiência mitral ( Figura 2 , A-C). Foram excluídos outros sinais de infecção localmente não controlada e estabelecido o diagnóstico de endocardite infecciosa.

Embora não tenha havido consenso sobre esquemas antibióticos adequados, o paciente recebeu terapia antimicrobiana com meropenem e gentamicina por seis e duas semanas, respectivamente. Atendendo à presença de neoplasia metastática ativa, não foi considerado candidato à intervenção cirúrgica.

Hemoculturas repetidas após três dias de antibioticoterapia eficaz foram negativas. A evolução clínica do paciente foi favorável, com redução progressiva do vasopressor e do suporte respiratório, permitindo a transferência para a Unidade Oncológica para conclusão do tratamento.

**Figura 3:**
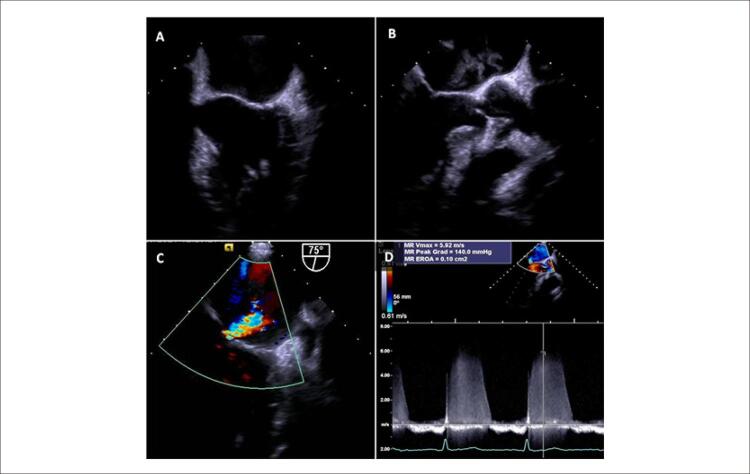
– Ecocardiografia transesofágica realizada no 51º dia de internação após antibioticoterapia. Revela eliminação completa da vegetação valvar mitral (visão zoom da valva mitral em 60º (A) e 120º (B)). Doppler colorido (C) e de onda contínua (D) revelando regurgitação valvar mitral leve (área efetiva do orifício regurgitante de 0,10 cm 2 pelo método PISA).

Na repetição da ETE no 51º dia de internação, a vegetação desapareceu, sendo visível apenas regurgitação mitral leve. Não foram observadas imagens sugestivas de fístula, abscesso ou formação de aneurisma ( Figura 3 , A-D). O paciente recebeu alta e manteve acompanhamento médico, embora tenha decidido não recorrer a esquemas quimioterápicos adicionais.

## Discussão

Os autores relatam um caso raro de endocardite infecciosa causada por *S. marcescens* , agente extremamente raro, responsável por 0,1% de todos os casos de endocardite. Historicamente, *S. marcescens* , um bacilo gram-negativo oportunista, tem sido considerado um agente causador de EI em usuários de drogas intravenosas, mas recentemente foram relatados alguns casos em não usuários de drogas com distúrbios médicos crônicos. ^6 - 8^

Em pacientes com dispositivos intravasculares e choque séptico, se deve manter um alto índice de suspeita para diagnosticar uma infecção relacionada ao dispositivo. ^2 , 3^ Em nosso caso, o cateter venoso central de longa permanência foi provavelmente a fonte da bacteremia no ambiente hospitalar.

A endocardite por *S.marcescens* frequentemente afeta as valvas do lado esquerdo, causando destruição significativa da valva, embolização séptica e altas taxas de mortalidade sem intervenção cirúrgica. ^6 , 7 , 9 , 10^ A terapia antimicrobiana apropriada é incerta devido à considerável resistência aos antibióticos, mas os regimes aceitos geralmente envolvem uma combinação de um betalactâmico e um aminoglicosídeo. ^4 , 5 , 8^ A cirurgia é frequentemente negada a pacientes com câncer, mal orientados pelo conceito de carga considerável de comorbidades e a terapia antitumoral geralmente é suspensa. ^1 , 2^

Neste caso, embora inesperado, a abordagem conservadora com antibioticoterapia foi bem-sucedida, resultando na resolução completa da vegetação mitral no ETE com sequelas mínimas e eliminando a necessidade de cirurgia.

Até onde sabemos, esta é a primeira terapia médica bem-sucedida relatada para EI causada por *S. marcescens* em um paciente imunocomprometido. Este caso ressalta importantes considerações da prática clínica. Pacientes com câncer em quimioterapia de longo prazo com acesso vascular enfrentam um risco aumentado de infecções da corrente sanguínea associadas aos cuidados de saúde, enfatizando a necessidade de cautela durante procedimentos invasivos. A apresentação clínica atípica requer alta suspeita para detecção e tratamento precoces. Dada a sua raridade, cada caso relatado na literatura contribui para uma melhor compreensão das estratégias ideais de prevenção e tratamento. ^9 , 10 - 12^

## References

[1] Cosyns B, Roosens B, Lancellotti P, Laroche C, Dulgheru R, Scheggi V (2021). Cancer and Infective Endocarditis: Characteristics and Prognostic Impact. Front Cardiovasc Med.

[2] Kim K, Kim D, Lee SE, Cho IJ, Shim CY, Hong GR (2019). Infective Endocarditis in Cancer Patients - Causative Organisms, Predisposing Procedures, and Prognosis Differ from Infective Endocarditis in Non-Cancer Patients. Circ J.

[3] Grable C, Yusuf SW, Song J, Viola GM, Ulhaq O, Banchs J (2021). Characteristics of Infective Endocarditis in a Cancer Population. Open Heart.

[4] Pugalenthi LS, Ahmad M, Reddy S, Barkhane Z, Elmadi J, Satish Kumar L (2022). Malignancy and Endocarditis: Divulging Into the Intertwined Association. Cureus.

[5] Mistiaen WP, Gebruers N (2020). How to Manage Patients in Whom Malignancy and Infective Endocarditis are Associated: A Review. Scand Cardiovasc J.

[6] Phadke VK, Jacob JT (2016). Marvelous but Morbid: Infective Endocarditis Due to Serratia Marcescens. Infect Dis Clin Pract (Baltim Md).

[7] Elkattawy S, Mohammadian M, Williams N, Mowafy A, Ayad S, Noori MAM (2021). Serratia Marcescens Endocarditis. Cureus.

[8] Goodberlet M, Schontz MJ, McLaughlin K, Kelly J (2019). Uncertainty of Treatment of Serratia marcescens Endocarditis. Int J Med Pharm.

[9] Ferreira AI, Oliveira ESF, Reis J, Henriques M, Almeida J (2022). Serratia Marcescens Endocarditis: A Case Report and Literature Review. Acta Med Port.

[10] Luttmann K, Starnes VR, Haddad M, Duggan J (2022). Serratia Marcescens, a Rare and Devastating Cause of Endocarditis: A Case Report and Review of the Literature. Cureus.

[11] Yeung HM, Chavarria B, Shahsavari D (2018). A Complicated Case of Serratia Marcescens Infective Endocarditis in the Era of the Current Opioid Epidemic. Case Rep Infect Dis.

[12] Singh A, Tappeta K, Chellapuram N, Singh D (2022). An Interesting Case of Serratia Endocarditis in a Patient with Chronic Myeloid Leukemia. Cureus.

