# Supplementary material for: Endocardite da Válvula Mitral – Uma Causa Rara num Doente com Neoplasia
Source: Arq Bras Cardiol. 2023 Oct 20;120(10):e20230268. [Article in Portuguese] doi: 10.36660/abc.20230268 (PMC10593386; doi:10.36660/abc.20230268)
Supplement: Translation

# Translation: Mitral Valve Endocarditis – An Unusual Culprit in a Cancer Patient

**Authors:** Fabiana Duarte, Carina Machado, Luis Oliveira, Duarte Machado, Raquel Dourado

**Affiliations:** 1 Portugal Hospital do Divino Espirito Santo de Ponta Delgada EPE – Cardiologia, Açores – Portugal

**Keywords:** Endocarditis, Drug Therapy, Echocardiography

## Introduction

Cancer patients are prone to infective endocarditis (IE), associated with specific causative agents, leading to poorer outcomes. The prevalence of IE in cancer patients ranges from 5.6% to 18%, with a reported 1-year mortality rate of nearly 30%. ^1 - 3^ Risk factors, such as a suppressed immune system, intravascular catheter use, and hypercoagulability, predispose malignancy patients to IE. ^2 , 3^

Clinical evolution is unfavorable due to high mortality rates related to antibiotic resistance, underlying medical conditions, lack of performed surgical interventions, and antitumoral therapy regimen discontinuation. ^2 - 5^

According to the literature, *Staphylococcal* species, mainly *Staphylococcus aureus* and Enterococci, are common microorganisms causing IE in cancer patients. ^2 , 3^

We describe an unusual case of mitral valve IE caused by *Serratia marcescens* in a patient with hematological malignancy. Although bacteremia caused by *S. marcescens* , a non-HACEK gram-negative bacillus, is not uncommon, its association with IE is quite rare. ^6 , 7^

### Case report

A 51-year-old male patient with a history of hypertension, smoking habits, and obesity underwent partial penectomy for ulcerated penile squamous cell carcinoma in January 2020. In March 2021, a thoracic-abdominopelvic computed tomography (CT) scan revealed a mass on the left of the inguinal region with ilioinguinal and para-aortic enlarged lymph nodes. A biopsy confirmed regional lymph node (LN) metastases of penile carcinoma, and after multidisciplinary discussion, the patient was proposed for neoadjuvant chemotherapy before LN resection. Three courses of chemotherapy were completed through an implantable long-term central venous access in the right subclavian vein. No immediate complications were reported.

Approximately 24 hours after the last treatment course, the patient presented to the emergency department complaining of fatigue, loss of appetite, and fever. The patient denied pleuritic pain or abdominal symptoms. On physical examination, he was hypotensive and febrile (tympanic temperature 38.4°C), with polypnea, hypoxemia, and temporal and spatial disorientation. No additional findings were reported, including unremarkable cardiac examination. Blood sample analysis revealed anemia (hemoglobin 9.8 g/dL) and thrombocytopenia (platelet count 100000/µL), acute kidney injury (creatinine 3.68 mg/dL, urea 130 mg/dL), rhabdomyolysis (creatine kinase 18567 U/L), high levels of C-reactive protein (CRP 19 mg/dL) and procalcitonin (PCT 51.6 ng/mL).

During the diagnostic work-up, a thoracic CT scan showed multiple lung solid and cavitary nodules suggestive of septic pulmonary emboli ( Figure 1 , A-C). At this stage, the diagnosis of septic shock with multiorgan dysfunction was proposed. Two sets of peripheral blood cultures were collected, and the patient was started on empirical antimicrobial therapy with ceftriaxone and metronidazole and was admitted to the Intensive Care Unit.

**Figure 1:** – A, B, C) Computed tomography chest scan (lung window) demonstrating bilateral wedge-shaped nodular lesions, some with cavitation (yellow arrow), the majority located at the periphery. Nodules have diameters ranging from a few millimeters to 18 mm, with the largest one being located on the left upper lobe.

His condition deteriorated, requiring high-dose norepinephrine vasopressor therapy and supplemental oxygen. An abdominal/pelvic CT scan revealed the progression of neoplastic disease, and a brain CT scan showed no signs of cerebral embolization.

According to antibiotic sensitivity tests *, Serratia marcescens* was isolated from blood cultures, and the initial antimicrobial regimen was adjusted to piperacillin-tazobactam. The long-term central venous catheter was removed and sent for culture, which isolated the same microorganism. A transthoracic echocardiogram was initially performed but was inconclusive due to a very poor acoustic window. A transesophageal echocardiography (TEE) performed during hospitalization revealed a single large mitral valve vegetation (maximum diameter of 23 mm) attached to the posterior leaflet of the mitral valve in the P2 and P3 scallop regions, without mitral regurgitation ( Figure 2 , A-C). Other signs of locally uncontrolled infection were excluded, and the diagnosis of infective endocarditis was established.

**Figure 2:** – Initial transesophageal echocardiography performed during hospital stay. It revealed a large, filiform and mobile mitral vegetation (maximum diameter of 23 mm), attached to the atrial surface of the posterior mitral leaflet, involving P2 and P3 scallop regions (yellow arrow); TEE longitudinal view at 60º (A) and at 120º (B). No scallops prolapse or mitral regurgitation were evident (C).

Although there was no consensus about appropriate antibiotic regimens, the patient received antimicrobial therapy with meropenem and gentamicin for six and two weeks, respectively. Attending to the presence of active metastatic neoplasm, he was not considered a candidate for surgical intervention.

Repeated blood cultures after three days of effective antibiotic therapy were negative. The patient´s clinical evolution was favorable, with progressive reduction of vasopressor and respiratory support, allowing for transfer to the Oncology Unit to complete the treatment.

On repeat TEE on the 51 ^st^ day of the hospital stay, the vegetation disappeared, and only mild mitral regurgitation was visible. No images suggestive of fistula, abscess, or aneurysm formation were observed ( Figure 3 , A-D). The patient was discharged and kept medical follow-up, although he decided against additional chemotherapy regimens.

**Figure 3:** – Transesophageal echocardiography performed at the 51 st day of hospital stay after antibiotic therapy. It reveals complete elimination of the mitral valve vegetation (zoom view of mitral valve at 60º (A) and 120º (B)). Color (C) and continuous (D) wave Doppler revealing a mild mitral valve regurgitation (effective regurgitant orifice area of 0.10 cm 2 by PISA method).

## Discussion

The authors report a rare case of infective endocarditis caused by *S. marcescens* , an extremely rare agent accounting for 0.1% of all cases of endocarditis. Historically, *S. marcescens* , an opportunistic gram-negative bacillus, has been considered a causative agent of IE in intravenous drug users, but recently, a few cases in non-drug users with chronic medical disorders were reported. ^6 - 8^

In patients with intravascular devices and septic shock, a high index of suspicion should be maintained to diagnose a device-related infection. ^2 , 3^ In our case, the long-term central venous catheter was probably the source of the bacteremia in the hospital setting.

*S. marcescens* endocarditis often affects left-sided valves, causing significant valve destruction, septic embolization, and high mortality rates without surgical intervention. ^6 , 7 , 9 , 10^ Appropriate antimicrobial therapy is uncertain due to considerable antibiotic resistance but accepted regimens typically involve a combination of a beta-lactam and an aminoglycoside. ^4 , 5 , 8^ Surgery is often denied to cancer patients misguided by the concept of considerable comorbidity burden, and antitumoral therapy is usually withdrawn. ^1 , 2^

In this case, although unexpected, a conservative approach with antibiotic therapy was successful, resulting in complete resolution of mitral vegetation on TEE with minimal sequels and eliminating the need for surgery.

To our knowledge, this is the first reported successful medical therapy for *S. marcescens* -caused IE in an immunocompromised patient. This case underscores important clinical practice considerations. Cancer patients on long-term chemotherapy with vascular access face an increased risk of healthcare-associated bloodstream infections, emphasizing the need for caution during invasive procedures. Atypical clinical presentation requires high suspicion for early detection and treatment. Given its rarity, each reported case in the literature contributes to better comprehending optimal prevention and treatment strategies. ^9 , 10 - 12^
